# Behaviour change techniques in cardiovascular disease smartphone apps to improve physical activity and sedentary behaviour: Systematic review and meta-regression

**DOI:** 10.1186/s12966-022-01319-8

**Published:** 2022-07-07

**Authors:** Kacie Patterson, Rachel Davey, Richard Keegan, Brea Kunstler, Andrew Woodward, Nicole Freene

**Affiliations:** 1 Health Research Institute, University of Canberra, Bruce, ACT 2617 Australia; 2 Research Institute for Sports and Exercise (UCRISE), Faculty of Health, University of Canberra, Bruce, ACT 2617 Australia; 3 BehaviourWorks Australia, Monash University, Clayton, Victoria 3168 Australia; 4 Faculty of Health, University of Canberra, Bruce, ACT 2617 Australia; 5 Physiotherapy, Faculty of Health, University of Canberra, Bruce, ACT 2617 Australia

**Keywords:** mHealth, Hypertension, Stroke, Bayesian meta-analysis, Action planning, Lifestyle modification

## Abstract

**Background:**

Smartphone apps are increasingly used to deliver physical activity and sedentary behaviour interventions for people with cardiovascular disease. However, the active components of these interventions which aim to change behaviours are unclear.

**Aims:**

To identify behaviour change techniques used in smartphone app interventions for improving physical activity and sedentary behaviour in people with cardiovascular disease. Secondly, to investigate the association of the identified techniques on improving these behaviours.

**Methods:**

Six databases (Medline, CINAHL Plus, Cochrane Library, SCOPUS, Sports Discus, EMBASE) were searched from 2007 to October 2020. Eligible studies used a smartphone app intervention for people with cardiovascular disease and reported a physical activity and/or sedentary behaviour outcome. The behaviour change techniques used within the apps for physical activity and/or sedentary behaviour were coded using the Behaviour Change Technique Taxonomy (v1). The association of behaviour change techniques on physical activity outcomes were explored through meta-regression.

**Results:**

Forty behaviour change techniques were identified across the 19 included app-based interventions. Only two studies reported the behaviour change techniques used to target sedentary behaviour change. The most frequently used techniques for sedentary behaviour and physical activity were *habit reversal* and *self-monitoring of behaviour* respectively. In univariable analyses, *action planning* (*β* =0.42, 90%CrI 0.07–0.78) and *graded tasks* (*β* =0.33, 90%CrI -0.04-0.67) each had medium positive associations with increasing physical activity. Participants in interventions that used either *self-monitoring outcome(s) of behaviour* (i.e. outcomes other than physical activity) (*β* = − 0.47, 90%CrI -0.79--0.16), *biofeedback* (*β* = − 0.47, 90%CrI -0.81--0.15) and *information about health consequences* (*β* = − 0.42, 90%CrI -0.74--0.07) as behaviour change techniques, appeared to do less physical activity. In the multivariable model, these predictors were not clearly removed from zero.

**Conclusion:**

The behaviour change techniques *action planning* and *graded tasks* are good candidates for causal testing in future experimental smartphone app designs.

**Supplementary Information:**

The online version contains supplementary material available at 10.1186/s12966-022-01319-8.

## Introduction

Smartphone applications (apps) have potential to be powerful modalities for behaviour change by leveraging their unique capabilities to facilitate learning, engagement and motivation [1]. The evolution of technology and smartphone ubiquity has seen smartphone app interventions as increasing attractive options for remote monitoring and supplements to usual healthcare. This is evident in a small but growing body of cardiovascular disease research, in particular for secondary prevention through lifestyle risk factor modification and reducing hospital readmissions [2–4].

Cardiovascular disease (CVD) is a leading cause of morbidity and mortality worldwide [5]. Two significant and independent risk factors impacting the progression of CVD and all-cause mortality are physical inactivity and sedentary behaviour [6]. Traditional secondary prevention is not always accessible or feasible with several system-level and patient-level barriers [7]. Therefore, flexible and innovative behaviour change interventions targeting these risk factors are important for secondary prevention. Thus, smartphone apps may be a suitable adjunct to traditional CVD healthcare.

Previous reviews show reasonable effectiveness of smartphone apps for increasing physical activity in older adults [8], adults without disease [9] and people with CVD [10]. These apps typically produce small to moderate effects. The impact of smartphone apps on sedentary behaviour for people with CVD is less clear with very few studies [10]. Due to the diverse features of smartphone apps it is unclear how these interventions are changing behaviour and is further complicated by the constant advances in technology. Therefore, further investigation into identifying the components that distinguish which approaches are most effective in increasing physical activity and decreasing sedentary behaviour is needed.

Identifying the specific active ingredients, or behaviour change techniques (BCTs), used in interventions is important. It allows for comparing and potentially replicating successful intervention components. The Behaviour Change Technique Taxonomy (v1) is one method for identifying these active ingredients in a standardized way [11]. Furthermore, analyses to investigate the moderating role of each BCT on physical activity and/or sedentary behaviour outcomes can help explain some of the between-study variation in effectiveness. An example of this was reported by Michie et al. [12] and found interventions for increasing physical activity and healthy eating were more effective when combining *self-monitoring* with one or more BCTs derived from control theory (e.g. *goal setting*, *review behaviour goal(s)*, *feedback on behaviour*). The moderating role of BCTs is yet to be explored in people with CVD using physical activity and sedentary behaviour smartphone apps.

The identification of BCTs used in other types of technology including phone calls, text-messaging, internet, and mobile sensors for increasing physical activity by people with CVD has already been reviewed in the literature [2, 13]. The BCTs *self-monitoring of behaviour*, *goal setting (behaviour)*, *social support (practical)*, *information about health consequences*, *feedback on performance*, and *prompts/cues* were frequently used [2, 13]. Nevertheless, the moderating role of each of these BCTs for CVD remains unknown. Despite limited studies exploring smartphone apps and sedentary behaviour in CVD, there is also benefit from examining the available literature to help shape the future direction of this work. Physical activity and sedentary behaviour pose independent health risks [6] and should be targeted with separately focused interventions [14, 15]. As such, BCTs may also need to be targeted to each behaviour.

Therefore, the aim of this review is to identify the BCTs included in smartphone app interventions used to increase physical activity and/or decrease sedentary behaviour in people with CVD. Secondly, this review aims to determine how these BCTs individually influence the effectiveness of apps on improving physical activity and sedentary behaviour.

## Methods

This systematic review was informed by the PRISMA statement (2020) [16] and the Cochrane Handbook for Systematic Reviews of Interventions [17]. The protocol was prospectively registered with PROSPERO (CRD42020189046). The methods have been described in full elsewhere in a prior meta-analysis of physical activity outcomes [10].

### Search strategy

Six electronic databases including Medline, CINAHL Plus with Full Text (EBSCO), Cochrane Library, SCOPUS, Sports Discus and EMBASE were searched. Appropriate keywords, subject headings, wild cards and truncations were used in relation to CVD (population group), smartphone apps (intervention), and physical activity and/or sedentary behaviour (key outcomes). Searches were conducted on 31 October 2020. Peer-reviewed, English language, full-text studies of any design were included since the launch of the first app stores in 2007. Reference lists of eligible studies and review articles were also screened.

### Study selection criteria

Experimental studies of all study designs were included provided they were conducted with people aged ≥18 years with CVD (including coronary heart disease, heart failure, hypertension, cerebral vascular disease (stroke), peripheral artery disease, rheumatic heart disease, congenital heart disease, cardiomyopathies and cardiac arrhythmias) and delivered a secondary prevention intervention through a smartphone or tablet computer app. Additionally, the app-based intervention must have targeted or measured physical activity and/or sedentary behaviour. Interventions which used only short messaging service (SMS), websites, video conferencing, telehealth or phone calls were excluded.

### Screening, data extraction and risk of bias

Covidence Systematic Review Software (Vertias Health Innovation, Melbourne, Australia, www.covidence.org) was used to screen titles and abstracts by two independent investigators using a priori screening criteria (KP and NF). Full-text articles were independently reviewed (KP and NF). Disagreements were discussed and resolved by consensus. The Template for Intervention Description and Replication (TIDieR) Checklist [18] was used to record information from the included studies by two investigators (KP and NF). Information was sourced from published appendices, protocols, supporting studies and results papers. Further information was extracted by one investigator (KP) and confirmed by a second (NF) including: study background; sample-related information; outcome-related information; and any behaviour change theories or models used.

Studies were assessed for risk of bias by two investigators (KP and NF) using the relevant tools: Revised Cochrane risk-of-bias (RoB-2) for randomized control trials [19], ROBINS-I tool for quasi-experimental and non-randomized control trials [20], and Quality Assessment Tool for Before-After (Pre-Post) Studies With No Control Group [21]. Risk of bias was classified as low, moderate or high for each study.

### Behaviour change technique coding

The BCTs were independently coded according to Michie et al.’s BCT Taxonomy v1 (93-item coding framework across 16 categories) [11] by two trained investigators (KP and BK). The TIDieR checklist was used to extract and consolidate key information about the app-based interventions for the coding of BCTs. Behaviour change techniques were coded as either being present or absent for each intervention and for app-based control interventions. A BCT was only coded as present where there was clear evidence of its direct application to either physical activity, sedentary behaviour or both and delivered via the app. A BCT could only be coded once per intervention. For BCTs related to outcomes of behaviour (e.g. *self-monitoring of outcome(s) of behaviour*), the BCT was coded if the outcome related to either physical activity or sedentary behaviour. For example, measuring blood pressure as an outcome related to exercise participation. BCTs were not coded where authors only mentioned their use without sufficient explanation about how the BCT was delivered. For example, if the authors mentioned the study was designed to create physical activity habits but did not explain the process of how, the BCT *habit formation* was not coded. Any discrepancies were resolved by discussion. The total number of BCTs used in each intervention, category and behaviour was counted.

Cohen’s kappa statistic (*κ*) was used to measure interrater reliability for BCT coding [22]. Interrater reliability was assessed for those techniques coded as being present by at least one coder. A *κ* of ≥0.81 is near perfect agreement, 0.61–0.80 substantial agreement, 0.41–0.60 moderate agreement, 0.21–0.40 fair agreement, 0.1–0.2 slight agreement, and 0 agreement equivalent to chance [22].

### Strategy for data synthesis

All studies were included in the narrative synthesis. The BCTs were classified as being frequently used for either physical activity or sedentary behaviour when identified in ≥50% of the interventions. Combinations of BCTs were also explored for patterns of frequent use between interventions (e.g. *goal setting* + *graded tasks* + *feedback on behaviour*).

### Data analysis and meta-regression

Controlled trials were included in the data analysis and meta-regression of physical activity and sedentary behaviour, provided there were three or more studies available. No meta-regression was possible for sedentary behaviour due to only two controlled trials being eligible for inclusion. Where physical activity was reported using more than one method within a trial and recorded using the same measurement tool, moderate-to-vigorous intensity was used as it was the most prevalent physical activity measure amongst all studies. Due to physical activity outcomes being assessed with a range of different measurement tools (e.g. self-report questionnaires, accelerometers) and units (e.g. steps per day, moderate-to-vigorous intensity physical activity minutes, meeting physical activity guidelines etc.), the standardized mean difference in post-study outcomes between the intervention and control group was calculated using an effect size calculator [23]. The between-group means, standard deviations and sample sizes were used to calculate the effect size. Effect sizes were interpreted according to Cohen’s guidelines: 0.20 small, 0.5 medium, 0.8 large, 1.3 very large [24]. One controlled trial [25] only provided the difference in pre-post means for the control group and therefore could not be included in the analysis.

Meta-regression modelling was conducted on the observed intervention standardized difference-in-means (*d*), with estimation of the association of BCTs on physical activity outcomes. Only BCTs present in two-or-more, and absent in two-or more studies were assessed to minimize identifiability issues. Meta-analytic models included a study-level ‘random’ intercept (random-effects meta-analysis). The analyses were implemented via the ‘brms’ package [26] in R statistical software, an interface to the Bayesian analysis language Stan [27]. Models were of the general form:

1 $$d\sim {\beta}_0+\left(\beta \cdotp BCT\left\{0,1\right\}\right)+N\left(0,\tau \right)$$

Where the heterogeneity had a normal prior distribution with mean 0, and standard deviation τ (standard deviation of the between-study variability). τ had a half-Cauchy prior *HC*(0, 1). The population intercept β_0_ had normal prior *N*(0, 1), and BCT effects *β* (dummy-coded) had Cauchy priors *C*(0, 1), intended to be weakly informative. Convergence was assessed using the Rhat statistic [28], effective sample size, and inspection of the MCMC chains. A separate analysis was conducted for each BCT, in univariable format. Interpretation of the BCT effects was conducted directly via the estimated coefficient β.

Secondly, a multivariable analysis was conducted across all BCTs. As the number of candidate predictors exceeded the number of observations, a variable selection strategy was applied. A regularized horseshoe prior [29] was selected for each BCT. This is a shrinkage prior that places a high probability at zero, expressing that only a minority of the parameters are believed to have a non-zero value. The expected proportion of non-zero coefficients was set as half, and the scale (the width of the non-zero component) to one. All BCTs were included simultaneously as binary predictors, as in the univariable analysis. No interaction terms were included and relationships between the BCT (collinearity) were not considered. For both analyses, the posterior distributions and credible intervals of the parameter estimates were visualized, as implemented in package ‘ggdist’ [30] via ‘ggplot2’ [31]. Sensitivity analyses were planned to remove studies at high risk of bias.

## Results

### Study selection and characteristics

**Fig. 1:**
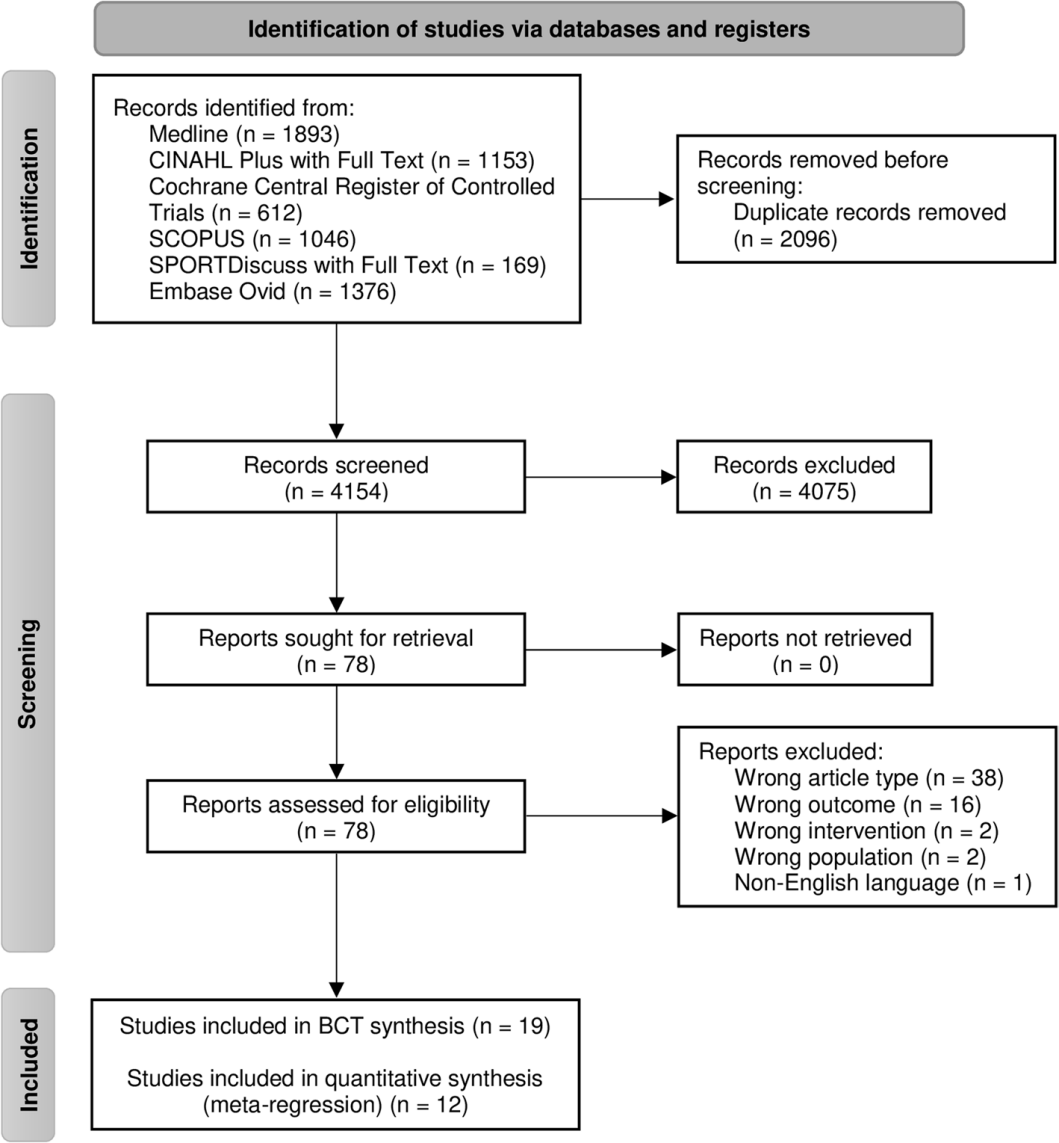
PRISMA flow diagram demonstrating the flow of studies through the review

Electronic searches identified 4154 studies after duplicates were removed (Fig. 1). Full-text screening was completed for 78 studies, with 19 studies included in the final review [25, 32–49]. Agreement between reviewers for coding BCTs was substantial (*κ* = 0.70).

Of the 19 included studies involving 1543 participants, the majority involved participants with coronary heart disease (*n* = 10) [25, 32, 34, 36, 38, 40, 44–46, 49], followed by four with hypertension [37, 39, 42, 47], three with stroke [35, 41, 43], one with heart failure [48], and one with peripheral artery disease [33] (Supplement 1). Four studies measured sedentary behaviour as an outcome [34, 35, 41, 45]. Sedentary behaviour outcomes were reported as sedentary or sitting time per day, and duration and number of sedentary bouts per day. All 19 studies reported a physical activity outcome such as steps per day, minutes of moderate-to-vigorous intensity physical activity, meeting American College of Sports Medicine guidelines or number of 30-minute physical activity sessions per week. Risk of bias was high for all randomized controlled trials (*n* = 10) [32, 33, 35–38, 42, 44, 46, 49] and non-randomized controlled trials (*n* = 3) [25, 41, 43]. The high risk of bias was primarily due to lack of blinding of outcome assessors and participants. Overall, the cohort studies (*n* = 6) [34, 39, 40, 45, 47, 48] were considered moderate risk of bias primarily due to small sample sizes and using self-report outcome measures. Additional risk of bias results are available elsewhere [10] and further descriptive results are in Supplement 1.

Behaviour change theories and models were mentioned to inform the development of app-based interventions in nine of the studies. These included motivational interviewing [32, 38, 47], transtheoretical theory [32, 38], health belief model [39, 44], cognitive behavioural therapy [42, 46], cognitive flexibility [34], theory of planned behaviour [39], social cognitive theory [39], control theory [41], FITT principles model [44], COM-B [47], and elaboration likelihood model [47].

### Behaviour change techniques identified in smartphone app interventions targeting physical activity and sedentary behaviour

Forty unique BCTs were identified across the 19 app-based interventions, totalling 260 BCTs (Table 1). A brief description and example of each of the BCTs is available in Supplement 2. These BCTs mostly belonged to the ‘feedback and monitoring’ category (30.7%, *n* = 80/260). *Self-monitoring of behaviour* and *adding objects to the environment* were the most frequently used BCTs and were used in all 19 apps. *Adding objects to the environment* was used in all interventions because of the provision of the smartphone app.

**Table 1:** Number of times each behaviour change technique was used

Behaviour change techniques	PA	SB	Other*	Total
**Feedback and monitoring**				**80**
Monitoring of behaviour by others without feedback	8	0	0	8
Feedback on behaviour ^a^	16	1	0	17
Self-monitoring of behaviour ^a^	19	0	0	19
Self-monitoring of outcome(s) of behaviour ^b^	0	0	10	10
Monitoring of outcome(s) of behaviour without feedback	2	0	4	6
Biofeedback ^a^	12	0	0	12
Feedback on outcome(s) of behaviour	8	0	0	8
**Goals and planning**				**40**
Goal setting (behaviour) ^a^	12	1	0	13
Problem solving	6	0	0	6
Goal setting (outcome)	0	0	4	4
Action planning	10	1	0	11
Review behaviour goal(s)	2	0	0	2
Discrepancy between current behaviour and goal	4	0	0	4
**Antecedents**				**23**
Restructuring the social environment	1	1	0	2
Adding objects to the environment ^a^	19	2	0	21
**Repetition and substitution**				**23**
Behavioural practice/rehearsal	2	1	0	3
Behaviour substitution	1	1	0	2
Habit formation	1	1	0	2
Habit reversal	2	2	0	4
Generalisation of target behaviour	1	0	0	1
Graded tasks	10	1	0	11
**Social support**				**19**
Social support (unspecified) ^a^	12	0	0	12
Social support (practical)	3	1	0	4
Social support (emotional)	3	0	0	3
**Comparison of outcomes**				**15**
Credible source ^a^	14	1	0	15
**Natural consequences**				**13**
Information about health consequences	9	0	0	9
Information about social and environmental consequences	2	0	0	2
Monitoring of emotional consequences	2	0	0	2
**Associations**				**13**
Prompts/cues ^a^	11	1	0	12
Reduce prompts/cues	1	0	0	1
**Shaping knowledge**				**13**
Instruction on how to perform the behaviour	10	1	0	11
Information about antecedents	1	1	0	2
**Reward and threat**				**11**
Non-specific reward	1	0	0	1
Social reward	9	0	0	9
Social incentive	1	0	0	1
**Comparison of behaviour**				**6**
Demonstration of the behaviour	5	0	0	5
Social comparison	1	0	0	1
**Identity**				**2**
Framing/reframing	1	0	0	1
Incompatible beliefs	1	0	0	1
**Regulation**				**2**
Conserving mental resources	1	1	0	2
**Total**	**224**	**18**	**18**	**260**

^a^Behaviour change techniques frequently used for physical activity in ≥50% of interventions.

^b^Behaviour change techniques frequently used for outcomes related to physical activity or sedentary behaviour in ≥50% of interventions.

*Other outcomes related to physical activity and/or sedentary behaviour.

Note: Behaviour change techniques are organized in descending order of category. The total frequency of each behaviour change technique is displayed for all interventions.

Abbreviations: *PA* Physical activity, *SB* Sedentary behaviour

Though four studies measured sedentary behaviour, only two studies [34, 45] reported the BCTs used to target sedentary behaviour change. The 18 BCTs reported as used in these two studies were also used to increase physical activity (Table 1). For example, feedback on sedentary behaviour and feedback on physical activity. The BCTs targeting sedentary behaviour mostly belonged to the ‘repetition and substitution’ category (33.3%, *n* = 6/18), with only *adding objects to the environment* and *habit reversal* being common to both studies.

Frequently used BCTs (BCTs identified in ≥50% of interventions) for increasing physical activity included *self-monitoring of behaviour* (100%, *n* = 19/19), *adding objects to the environment* (100%, n = 19/19), *feedback on behaviour* (84.2%, *n* = 16/19), *credible source* (73.7%, *n* = 14/19), *goal setting (behaviour)* (63.2%, *n* = 12/19), *biofeedback* (63.2%, n = 12/19), *social support (unspecified)* (63.2%, n = 12/19), and *prompts/cues* (57.9%, n = 11/19) (Table 1). *Self-monitoring of outcome(s) of behaviour* (52.6%, n = 10/19) was used frequently for other outcomes related to physical activity and sedentary behaviour such as blood pressure or weight monitoring (Table 1).

None of the studies, including those using the same behavioural theory or framework, tested the same combination of BCTs. Therefore, it was not possible to isolate a set combination of BCTs and test them further for effectiveness in meta-regression analyses. Studies which had a medium to large positive effect on increasing physical activity [32, 35, 38, 41, 44, 49] (Supplement 1), all used *self-monitoring of behaviour* and *adding objects to the environment*, and at least three of the following BCTs: *action planning*, *monitoring of behaviour by others without feedback*, *feedback on behaviour*, *prompts/cues*, *graded tasks* and *credible source*. In contrast, there was no clear pattern of BCTs used in studies with small positive or negative effects on increasing physical activity [33, 36, 37, 42, 43, 46]. The median number of BCTs used was similar for studies with a positive medium to large effect (median = 11, range 6–19) and those with a small or negative effect (median = 11, range 7–15) on increasing physical activity.

In contrast to the controlled trials, cohort studies [34, 39, 40, 45, 47, 48] included a median number of 16 BCTs (range 7–22). The majority of cohort studies used the BCTs *goal setting (behaviour)* (*n* = 5/6), *feedback on behaviour* (n = 5/6), *self-monitoring of outcome(s) of behaviour* (n = 5/6), *biofeedback* (n = 5/6), *feedback on outcome(s) of behaviour* (n = 5/6), *social support (unspecified)* (*n* = 5/6), *credible source* (*n* = 5/6), *instruction on how to perform the behaviour* (*n* = 4/6), *demonstration of the behaviour* (n = 4/6), and *graded tasks* (n = 4/6) (Supplement 1). These BCTs focus on different types of feedback belonging to the ‘feedback and monitoring’ category.

### Moderating effects of behaviour change techniques

**Fig. 2:**
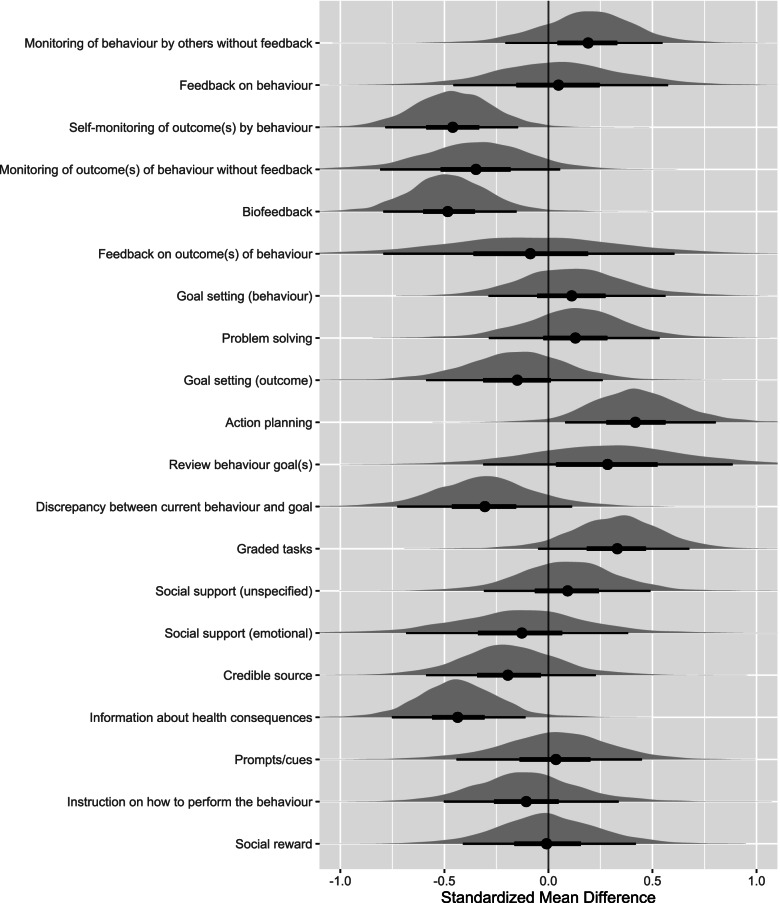
Univariable model distribution plot of behaviour change techniques. In this plot, the points are the estimated medians, the heavy bars are the 50% credible intervals and the light bars are the 90% credible intervals. The behaviour change techniques are listed based on behaviour change technique category

Twenty univariable analyses, including 12 controlled trials, were conducted to investigate differences in physical activity pooled effect size according to the presence of BCTs (Table 2 and Fig. 2). The BCTs towards the top of Table 2 largely focus on the practical skills of behaviour change belonging to the ‘goals and planning’, ‘feedback and monitoring’, ‘repetition and substitution’ and ‘social support’ categories. Of note, is the medium, positive association of *action planning* (*β* = 0.42, 90% CrI 0.07–0.78). This indicates that on average, studies which included *action planning* (*n* = 7/12) in the smartphone app compared to those that did not, differed in effect on improving physical activity by an additional 0.42. *Graded tasks* similarly had a medium, positive association (n = 6/12, *β* = 0.33, 90% CrI -0.04-0.67). The BCTs at the bottom of Table 2 largely focus on indirect monitoring and provision of information; belonging to the categories ‘feedback and monitoring’ and ‘natural consequences’. The BCTs *self-monitoring of outcome(s) of behaviour* (*n* = 5/12, *β* = −0.47, 90% CrI -0.79--0.16), *biofeedback* (*n* = 7/12, *β* = −0.47, 90% CrI -0.81--0.15) and *information about health consequences* (*n* = 6/12, *β* = − 0.42, 90% CrI -0.74--0.07) each appeared to have medium, negative associations on physical activity when included in smartphone apps for people with CVD. In addition, it appears the BCTs *action planning* and *self-monitoring of outcome(s) of behaviour* and *biofeedback* are collinear (Supplement 3).

**Table 2:** Results from univariable meta-regression analysis exploring moderating effects of behaviour change techniques on physical activity outcomes

Behaviour change techniques	Univariable meta-regression			
	n	*β* (90% CrI)	Intercept	*τ* (90% CrI)
Action planning	7	0.42 (0.07–0.78)*	0.07	0.22 (0.03–0.46)
Graded tasks	6	0.33 (−0.04–0.67)	0.18	0.21 (0.02–0.49)
Review behaviour goal(s)	2	0.27 (−0.33–0.89)	0.28	0.29 (0.06–0.55)
Monitoring of behaviour by others without feedback	6	0.18 (−0.22–0.54)	0.19	0.26 (0.03–0.54)
Problem solving	4	0.13 (− 0.28–0.54)	0.25	0.30 (0.07–0.58)
Goal setting (behaviour)	6	0.11 (−0.30–0.53)	0.25	0.32 (0.09–0.59)
Social support (unspecified)	6	0.11 (−0.29–0.51)	0.26	0.31 (0.08–0.59)
Feedback on behaviour	10	0.06 (−0.46–0.57)	0.27	0.32 (0.07–0.61)
Prompts/cues	7	0.02 (−0.42–0.44)	0.29	0.31 (0.08–0.59)
Social reward	5	−0.01 (− 0.43–0.41)	0.31	0.31 (0.08–0.59)
Feedback on outcome(s) of behaviour	2	−0.10 (− 0.80–0.60)	0.31	0.30 (0.08–0.57)
Instruction on how to perform behaviour	5	−0.11 (− 0.50–0.30)	0.35	0.30 (0.07–0.57)
Goal setting (outcome)	3	−0.12 (− 0.54–0.30)	0.36	0.32 (0.09–0.60)
Social support (emotional)	2	−0.14 (− 0.68–0.35)	0.34	0.32 (0.11–0.59)
Credible source	8	−0.20 (− 0.61–0.20)	0.43	0.28 (0.05–0.55)
Discrepancy between current behaviour and goal	2	−0.31 (− 0.72–0.09)	0.40	0.26 (0.04–0.52)
Monitoring of outcome(s) of behaviour without feedback	4	−0.35 (− 0.77–0.04)	0.40	0.27 (0.06–0.53)
Information about health consequences	6	−0.42 (− 0.74--0.07)*	0.56	0.16 (0.01–0.40)
Self-monitoring of outcome(s) of behaviour	5	− 0.47 (− 0.79--0.16)*	0.54	0.16 (0.02–0.38)
Biofeedback	7	−0.47 (− 0.81--0.15)*	0.60	0.15 (0.01–0.39)

Note: *Coefficient has a 90% credible interview excluding zero

Abbreviations: *n* number of studies, *β* regression coefficient, *CrI* credible interval, τ the standard deviation of the between-study variation (heterogeneity)

**Fig. 3:**
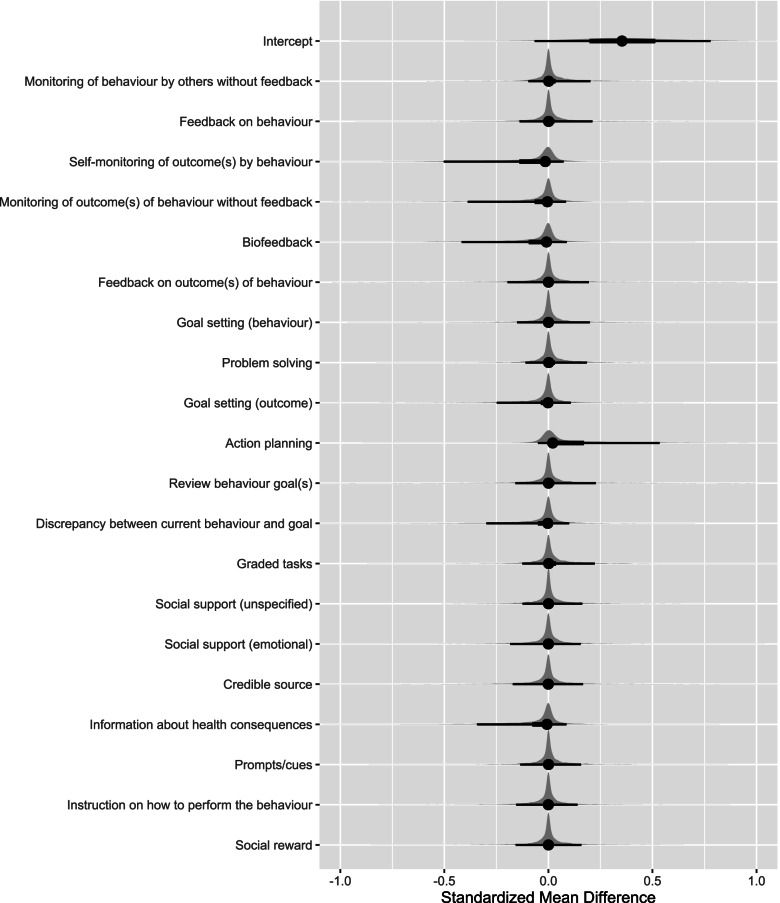
Multivariable model distribution plot of behaviour change techniques. In this plot, the points are the estimated medians, the heavy bars are the 50% credible intervals and the light bars are the 90% credible intervals. The behaviour change techniques are listed based on behaviour change technique category

Results of the multivariable analysis are shown in Table 3 and Fig. 3. There was a lack of evidence of an effect distinguished from zero for all BCT predictors, including those which appeared important in the univariable model. Sensitivity analyses were not performed as the risk of bias for all included studies was high.

**Table 3:** Results from multivariable meta-regression analysis exploring moderating effects of behaviour change techniques on physical activity outcomes

Behaviour change techniques	Multivariable meta-regression			
	*β*	Intercept	90% CrI	*τ* (90% CrI)
**Intercept**		0.36	−0.09 to 0.79	0.20 (0.02–0.48)
Monitoring of behaviour by others without feedback	0.02		−0.11 to 0.21	
Feedback on behaviour	0.02		−0.15 to 0.25	
Self-monitoring of outcome(s) of behaviour	−0.09		−0.48 to 0.07	
Monitoring of outcome(s) of behaviour without feedback	−0.06		−0.44 to 0.09	
Biofeedback	−0.07		−0.45 to 0.08	
Feedback on outcome(s) of behaviour	0.01		−0.19 to 0.19	
Goal setting (behaviour)	0.01		−0.15 to 0.19	
Problem solving	0.01		−0.13 to 0.19	
Goal setting (outcome)	−0.03		−0.25 to 0.11	
Action planning	0.10		−0.05 to 0.50	
Review behaviour goal(s)	0.01		−0.17 to 0.20	
Discrepancy between current behaviour and goal	−0.05		−0.37 to 0.10	
Graded tasks	0.02		−0.11 to 0.22	
Social support (unspecified)	0.01		−0.12 to 0.17	
Social support (emotional)	−0.01		−0.19 to 0.16	
Credible source	−0.01		−0.19 to 0.17	
Information about health consequences	−0.05		− 0.36 to 0.10	
Prompts/cues	0.01		−0.14 to 0.17	
Instruction on how to perform behaviour	−0.01		−0.16 to 0.15	
Social reward	0.01		−0.16 to 0.16	

Abbreviations: *β* regression coefficient, *CrI* credible interval, τ the standard deviation of the between-study variation (heterogeneity)

## Discussion

This review identified multiple BCTs used frequently in smartphone app physical activity and sedentary behaviour change interventions for people with CVD. Overall, all studies used *self-monitoring of behaviour* and *adding objects to the environment* due to the use of the app itself. There appears to be a medium, positive association of increased physical activity when *action planning* or *graded tasks* are incorporated in the smartphone app interventions for people with CVD. In addition, the BCTs *self-monitoring of outcome(s) of behaviour*, *biofeedback* and *information about health consequences* appear to have a medium, negative association with increasing physical activity when included. Meaning, participants in interventions that used either of these three techniques appeared to do less physical activity. In the multivariable analysis, all BCT predictors did not clearly show an association with increasing physical activity. Specific conclusions regarding BCTs for reducing sedentary behaviour were not possible due to the low number of included studies.

### Sedentary behaviour

*Habit reversal* was common to both studies aiming to reduce sedentary behaviour and was not used to target physical activity. A proportion of sedentary behaviour is habitual, meaning that it requires little to no conscious decision making [50]. Consistent with this review, this may mean that BCTs from the ‘repetition and substitution’ category may be needed to better control sedentary behaviours [14]. Gardner et al. [51] draw attention to the idea of while habitual behaviour may be discontinued or ‘broken’, the underlying habit association may remain. In turn, there poses risk of returning to the unwanted behaviours in moments lacking motivation or stress. Further, three BCTs have been highlighted as being suited to disrupting habit associations [51]. The included studies exploring sedentary behaviour [34, 45], included these techniques: *habit reversal, habit formation* and *behavioural substitution*. Additional investigation is warranted to explore habit association and these BCTs in sedentary behaviour change smartphone apps.

### Positive association on physical activity

The BCTs that had larger positive associations with increasing physical activity focused on the practical skills of behaviour change and belonged to the ‘goals and planning’, ‘feedback and monitoring’, ‘repetition and substitution’ and ‘social support’ categories. The BCTs included in these categories are consistent with reviews in eHealth interventions in CVD [13] and non-CVD populations [9, 52]. Examples of these practical skills for behaviour change include *action planning* which involves the prompt detailed planning of the behaviour including context, frequency, duration and intensity and *graded tasks* which progressively sets easy-to-perform tasks and builds them up until the desired behaviour is reached [11]. Further, the BCTs that are included in the above categories (e.g. *review behaviour goal(s)*) align with control theory and motivational interviewing techniques which are shown to be effective in increasing physical activity [12]. These BCTs incorporate self-regulatory techniques and involve feedback loops connected to a range of behavioural theories [53] which may help people with CVD address barriers to participating in physical activity. The BCTs and identified behavioural theories from the included studies appear to highlight the intervention developers’ assumptions that the reason people with CVD are not active is due to decision and motivational processes around readiness and overcoming barriers. The observations of this review suggest these BCTs (i.e. *action planning*, *graded tasks*, and *review behaviour goal(s)*) are good candidates for causal testing in future experimental smartphone app designs. This has been completed in an experimental design of general population, testing the presents versus absents *action planning*, *coping planning*, and *self-monitoring* in a self-regulation based e- and mHealth intervention to improve physical activity and sedentary behaviour [54]. Using the combination of all three BCTs was most effective at increasing physical activty and *action planning* only increased physical activity when used in combination with *coping planning* [54]. A similar experimental design would be beneficial with CVD participants.

### Negative association on physical activity

Monitoring behavioural outcomes and physiologic responses that were not directly related to physical activity (e.g. blood pressure) as a strategy to improve adoption of phyisical activity were key features of the BCTs that appeared to be negatively associated with physical activity outcomes. For example, *biofeedback*, *self-monitoring of outcome(s) of behaviour*, and *information about health consequences*. This may be explained as follows: when the focus is on the outcomes of behaviour, such as weight loss or blood pressure recordings, the attention shifts away from the behaviour itself (i.e. physical activity). This explanation is consistent with previous evidence noting interventions are most effective when the behaviour of interest is specifically targeted [12]. When self-monitoring focuses on the behaviour itself (i.e. physical activity), people with CVD can significantly improve physical activity [55].

### Strengths and limitations

This review has multiple strengths. The identification of BCTs using an established taxonomy facilitated comparisons between interventions and may contribute to the future development of apps using the identified BCTs associated with increasing physical activity. Furthermore, this review adds to the small but emerging literature by analysing the BCTs used in smartphone apps for sedentary behaviour change. However, important considerations must be made. Firstly, the risk of bias was high for the majority of included studies and hence the interpretation of the effect of intervention warrants caution. Secondly, the coding of BCTs was based upon the written information provided in the article and supporting materials which may have not been a complete description. When coding BCTs, clear evidence is required to confirm how the particular technique was applied. This is a valuable tool when publishing intervention descriptions as it establishes a common language. It also provides an additional layer for analysis when comparing one intervention to another and identifying possible explanatory factors for why a result may be different at the intervention level. Thirdly, analyses to investigate the association of BCTs for decreasing sedentary behaviour were not possible. When considering the completed analyses, a BCT that did not appear to have an association does not necessarily mean the technique has no effect on physical activity [52]. The effect may have been suppressed by another BCT used in combination or may require the presence of another BCT for the effect to be noted. Likewise, those identified as having an association cannot necessarily ensure greater increases in physical activity. This can be due to chance or related to other contextual factors and characteristics within each intervention which are beyond the scope of this review. Most app-based interventions are complex and ultimately their effectiveness may be determined by the level of engagement. These analyses also do not address the potential for simultaneous or collinear effects. It is difficult to isolate BCTs particularly when implemented in varying combinations, any of which may result in differing interaction effects likely resulting in omitted variable bias [52]. Additionally, by attempting to isolate BCTs they may become decontextualised from the overall intervention. Further, from the current review, it remains unclear whether set combinations of BCTs used in smartphone apps are more effective than others at increasing physical activity for people with CVD. Future research would benefit from experimental design, testing combinations of BCTs that appear favourable, such as *action planning* and *graded tasks*, for increasing physical activity in smartphone app interventions for people with CVD. Lastly, only 40 BCTs of the possible 93 BCTs were identified in the included studies. There is potential for other BCTs not tested in this context, being effective at changing physical activity and/or sedentary behaviour for being with CVD.

## Conclusions

Future development of smartphone apps for physical activity change among people with CVD would benefit from specifically testing the use of *action planning* and *graded tasks* in isolation or combination. Using these techniques may lead to potential clinical benefits as they involve a greater focus on the direct practical skills required for behaviour change. Caution may be warranted when using *self-monitoring outcome(s) of behaviour*, *biofeedback* and *information about health consequences* however experimental study designs would be required to evaluate the causal nature of any apparent associations. Smartphone apps for sedentary behaviour change is an emerging area showing potential however requires further exploration.

## Supplementary Information

**Additional file 1: Supplement 1.** Summary of studies

**Additional file 2: Supplement 2.** Behaviour change technique descriptions and examples from included studies

**Additional file 3: Supplement 3.** Grid plot of behaviour change techniques and studies ranked by observed effect size

## Data Availability

The dataset supporting the conclusions of this article is included within the article and its additional files.

## Abbreviations

Apps: Applications
BCT: Behaviour change technique
CVD: Cardiovascular disease
