# Supplementary material for: Behaviour change techniques in cardiovascular disease smartphone apps to improve physical activity and sedentary behaviour: Systematic review and meta-regression
Source: Int J Behav Nutr Phys Act. 2022 Jul 7;19:81. doi: 10.1186/s12966-022-01319-8 (PMC9261070; doi:10.1186/s12966-022-01319-8)
Supplement: Supplementary file 1 — Additional file 1: Supplement 1. Summary of studies [file 12966_2022_1319_MOESM1_ESM.docx]

**Supplement 1: Summary of studies**

| **Study** | **Participants** | **Intervention** | **Outcomes** | **Behaviour change techniques** | **Effect on increasing physical activity** |
| --- | --- | --- | --- | --- | --- |
| Duscha, 2018, Am Heart J, 199:105-114    RCT, USA | n = 25 (Exp 16, Con 9)  Age (yr) = Exp 59 (SD 8.1), Con 66.5 (SD 7.2)  F (%) = 24%  Dx = MI ± revascularization (PCI or CABG), valve repair, heart failure or stable angina  Setting = Outpatient phase-II CR | 12-weeks  Target behaviour = PA  Exp = Vida Health app and commercial Fitbit app – Fitbit Charge tracking + health coaching  Intensity = Weekly prompts  Purpose = Sustain PA patterns and monitor daily steps  Con = Usual care – no specific lifestyle recommendations from study personnel | Device-measured (Fitbit): steps.day^-1^, low PA mins.day^-1^, low PA mins.week^-1^, moderate-low PA mins.day^-1^, moderate-low PA mins.week^-1^, moderate-high PA mins.day^-1^, moderate-high PA mins.week^-1^, moderate PA mins.week^-1^, total active mins.day^-1^, total active mins.week^-1^  Follow up = Baseline, 12-weeks | Goal setting (behaviour), problem solving, action planning, review behaviour goal(s), feedback on behaviour, self-monitoring of behaviour, instruction on how to perform the behaviour, information about health consequences, prompts/cues, graded tasks, credible source, adding objects to the environment | Outcome measure: MVPA mins.week^-1^  Effect size: 1.046  Standard error: 0.442 |
| Duscha, 2018, Am J Cardiol, 122(5):879-884    RCT, USA | n = 20 (Exp 10, Con 10)  Age (yr) = 69 (SD 8.4)  F (%) = 15.8%  Dx = PAD with intermittent claudication who were sedentary  Setting = Outpatient cardiology clinic | 12-weeks  Target behaviour = PA  Exp = Commercial Fitbit app + Fitbit Charge activity tracker + web-based PAD patient information book  Intensity = Phone call if did not meet the prescribed steps for 2 consecutive weeks + weekly email with PAD tip of the week  Purpose = Monitor daily steps  Con = Hardcopy of PAD information book and physician guidance – no specific lifestyle recommendations from study personnel | Device-measured (Fitbit): steps.day^-1^, low PA mins.day^-1^, low PA mins.week^-1^, moderate-low PA mins.day^-1^, moderate-low PA mins.week^-1^, moderate-high PA mins.day^-1^, moderate-high PA mins.week^-1^, moderate PA mins.week^-1^, total active mins.day^-1^, total active mins.week^-1^  Baseline, 12-weeks | Goal setting (behaviour), action planning, review behaviour goal(s), feedback on behaviour, self-monitoring of behaviour, graded tasks, adding objects to the environment | Outcome measure: MVPA mins.week^-1^  Effect size: 0.177  Standard error: 0.460 |
| Freene, 2020, JMIR Form Res, 4(11):e17359    Pre-Post, Australia | n = 20  Age (yr) = 54 (SD 13)  F (%) = 15%  Dx = CR participants including stable CHD, CABG, PCI, MI  Setting = Outpatient phase-II CR | 6-weeks  Target behaviour = SB  Exp = Vire app and ToDo-CR behaviour change program + Fitbit Flex + GPS data  Intensity = Automatic messages generated from a bank by the system using machine learning. 14-19 push notifications during the 6-week intervention  Purpose = Self-monitoring activity levels and improve behavioural flexibility to change SB  Con = N/A | Device-measured (accelerometer – Actigraph ActiSleep): MVPA mins.day^-1^, LPA mins.day^-1^, SB mins.day^-1^, % SB.day^-1^, duration of SB bounts.day^-1^(mins), number of SB bouts.day^-1^, number of SB breaks.day^-1^, vector magnitude counts.day^-1^, steps.day^-1^  Baseline, 6-weeks, 16-weeks | Goal setting (behaviour), action planning, discrepancy between current behaviour and goal, feedback on behaviour, self-monitoring of behaviour, feedback on outcome(s) of behaviour, social support (unspecified), social support (practical), instruction on how to perform the behaviour, information about antecedents, information about health consequences, prompts/cues, behavioural practice/rehearsal, behaviour substitution, habit formation, habit reversal, graded tasks, credible source, social reward, conserving mental resources, restructuring the social environment, adding objects to the environment | N/A |
| Grau-Pellicer, 2020, Top Stroke Rehabil, 27(5):354-368    RCT, Spain | n = 41 (Exp 24, Con 17)  Age (yr) = Exp 62.96 (SD 11.87), Con 68.53 (SD 11.53)  F (%) = 48.7%  Dx = Chronic stroke  Setting = Community dwelling | 8-weeks  Target behaviour = PA (community ambulation) + SB  Exp = Fitlab Training app and Fitlab Test app including GPS and accelerometer monitoring + WhatsApp  Intensity = Unclear  Purpose = Supervise adherence to PA guidelines by monitoring walking distance and speed  Con = Usual care – conventional 3-month face-to-face rehabilitation program | Self-report: community ambulation mins.day^-1^, sitting time hours.day^-1^  Baseline, 3-months | Action planning, monitoring of behaviour by others without feedback, feedback on behaviour, self-monitoring of behaviour, biofeedback, social support (unspecified), monitoring of emotional consequences, credible source, adding objects to the environment | Outcome measure: MVPA mins.week^-1^  Effect size: 0.952  Standard error: 0.443 |
| Johnston, 2016, Am Heart J, 178:85‐94    Multicenter RCT, Sweden | n = 166 (Exp 86, Con 80)  Age (yr) = Exp 56.8 (SD 8), Con 58.4 (SD 8.6)  F (%) = 19%  Dx = MI  Setting = Traditional secondary prevention care including CR | 6-months  Target behaviour = Medication adherence + achievement of secondary prevention targets  Exp = SUPPORT app = e-diary for drug adherence + interactive patient support tool for information modules and recording data  Intensity = Every second day during the first 2 weeks, then 3 messages per week  Purpose = Monitor drug adherence and lifestyle behaviours including exercise, weight management and smoking  Con = Simplified smartphone drug adherence e-diary = report daily use of tricagrelor (medication) without feedback or education modules. Generic SMS if missed a dose. | Self-report (unspecified questionnaire): MPA mins.week^-1^, number of PA sessions.week^-1^, % exercise >150 min.week^-1^  Baseline, 6-months | Goal setting (behaviour), goal setting (outcome), discrepancy between current behaviour and goal, feedback on behaviour, self-monitoring of behaviour, self-monitoring of outcome(s) of behaviour, biofeedback, instruction on how to perform the behaviour, information about health consequences, reduce prompts/cues, credible source, social reward, adding objects to the environment | Outcome measure: MVPA mins.week^-1^  Effect size: -0.097  Standard error: 0.163 |
| Kim, 2016, JMIR, 18(6):e116    Sub-study of RCT, USA | n = 95 (Exp 52, Con 43)  Age (yr) = 57.6 (SD 8.6)  F (%) = 68%  Dx = Hypertension  Setting = Community dwelling accessing health facilities | 6-months  Target behaviour = Hypertension control- frequency of the use of alcohol, smoking, and exercise  Exp = HealthyCircles app = Wireless self-monitoring program + disease management + Withings Blood Pressure Monitor  Intensity = Encouraged to use 3x per week and take 2 measurements per day. If the participant did not meet the frequency for 2 weeks, a reminder email was sent  Purpose = Wireless self-monitoring of BP and education  Con = Usual care – HealthComp disease management program  Both = HealthComp disease management program with HealthComp relaying medical education | Self-report (Godin Leisure-time Exercise Questionnaire): weekly leisure activity score  Baseline, 6-months | Feedback on behaviour, self-monitoring of behaviour, self-monitoring of outcome(s) of behaviour, monitoring of outcome(s) of behaviour without feedback, biofeedback, social support (unspecified), information about health consequences, prompts/cues, credible source, adding objects to the environment | Outcome measure: Godin Leisure-time Exercise Activity score  Effect size: -0.057  Standard error: 0.206 |
| Lunde, 2020, Eur J Prev Cardiol, 27(16):1782-1792    Multicenter RCT, Norway | n = 113 (Exp 57, Con 56)  Age (yr) = 59 (SD 8.7)  F (%) = 22.1%  Dx = Heart disease including 73.4% CAD, 16.8% valve surgery and 9.8% other heart diseases  Setting = Varying CR programs across inpatient and outpatients | 12-months  Target behaviour = PA  Exp = Vett app = activity monitoring and feedback + goal setting + communicate with supervisor  Intensity = Motivational feedback 1-3x per week based on individual preference  Purpose = Set goals and reminders related to health behaviours  Con = Usual care – general advice according to a heart-friendly lifestyle and follow-up by their general practitioner | Self-report (interview): number of 30min moderate-to-vigorous intensity exercise sessions per week  Baseline, 12-months | Goal setting (behaviour), problem solving, goal setting (outcome), action planning, monitoring of behaviour by others without feedback, feedback on behaviour, self-monitoring of behaviour, social support (unspecified), prompts/cues, graded tasks, social reward, adding objects to the environment | Outcome measure: number of 30min moderate-to-vigorous intensity exercise sessions per week  Effect size: 0.630  Standard error: 0.203 |
| Lv, 2017, JMIR, 19(9):e311    Pre-Post, USA | n = 149  Age (yr) = 62.2 (SD 9.5)  F (%) = 51%  Dx = Hypertension  Setting = Ambulatory healthcare system | 6-months  Target behaviour = PA  Exp = EMPOWER-H app + Numera app + Web-based dashboard + Nurse Care Manager + wireless BP cuff, pedometer  Intensity = Instructed to measure and upload BP 2x day for at least 3 days per week and upload daily step count  Purpose = Support timely patient-provider interaction and personalized feedback for chronic disease management  Con = N/A | Self-report (Stanford Exercise Behaviour Scale): mins.week^-1^ aerobic exercise, mins.week^-1^ stretching or strengthening  Baseline, 6-months | Goal setting (behaviour), problem solving, goal setting (outcome), feedback on behaviour, self-monitoring of behaviour, self-monitoring of outcome(s) of behaviour, biofeedback, social support (unspecified), social support (practical), instruction on how to perform the behaviour, information about health consequences, information about social and environmental consequences, demonstration of the behaviour, graded tasks, credible source, social reward, adding objects to the environment | N/A |
| Nabutovsky, 2020, Israel Med Assoc J, 22(6):357-363    Pre-Post, Israel | n = 22  Age (yr) = 52.7 (SD 5.5)  F (%) = 22.7%  Dx = CAD  Setting = Outpatient cardiac prevention and rehabilitation centre | 6-months  Target behaviour = PA  Exp = Tele-CR with Datos Health app + care-team dashboard + smartwatch with Polar application  Intensity = Tele-health specialist consultation weekly via the messaging system or phone call. Exercise program updated every 5-weeks  Purpose = Remote monitoring, communication and management for secondary prevention  Con = N/A | Device-measured (smartwatch with  matching smartphone Polar application - Polar Inc, M430; Kempele,  Finland): steps.day^-1^, aerobic exercise mins.week^-1^, frequency of aerobic exercise.week^-1^, frequency of resistance exercise.week^-1^, attainment of 150min.week^-1^ of aerobic exercise  Baseline, 6-months | Goal setting (behaviour), action planning, feedback on behaviour, self-monitoring of behaviour, self-monitoring of outcome(s) of behaviour, biofeedback, feedback on outcome(s) of behaviour, social support (unspecified), instruction on how to perform the behaviour, demonstration of the behaviour, prompts/cues, generalisation of target behaviour, graded tasks, credible source, adding objects to the environment | N/A |
| Paul, 2016, Top Stroke Rehabil, 23(3):170‐177    Non-RCT, Scotland | n = 24 (Exp 16, Con 8)  Age (yr) = 56 (SD 10)  F (%) = 52%  Dx = Stroke  Setting = Stroke support groups | 6-weeks  Target behaviour = PA  Exp = STARFISH mobile phone app which users’ PA is visualized by fish swimming in a tank in virtual groups of 4  Intensity = Weekly increase in step goal  Purpose = Behavioural change intervention to encourage the user to become more physically active  Con = Usual care – no active rehabilitation, only appointments with health care professionals as required | Device-measured (accelerometer – Actigraph ActivPAL): steps.day^-1^, sedentary time hours.day^-1^, upright time hours.day^-1^, walking time hours.day^-1^  Baseline, 6-weeks | Goal setting (behaviour), action planning, feedback on behaviour, self-monitoring of behaviour, social support (unspecified), social comparison, graded tasks, social reward, social incentive, adding objects to the environment | Outcome measure: steps.day^-1^  Effect size: 1.023  Standard error: 0.463 |
| Persell, 2020, JAMA Network Open, 3(3):e200255    RCT, USA | n = 333 (Exp 166, Con 167)  Age (yr) = 58.9 (SD 12.8)  F (%) = 61.3%  Dx = Uncontrolled hypertension  Setting = Community dwelling receiving care from outpatient clinics | 6-months  Target behaviour = Hypertension control – PA, diet, medication adherence, BP measurement, sleep, stress management  Exp = Hypertension Personal Control Program (HPCP) = Artificial Intelligence (AI) technology + HPCP coaching app + blood pressure monitor  Intensity = Prompts daily BP measurement in the first week, then weekly prompts thereafter. Unspecified frequency of reminders for medication, PA, weight and diet  Purpose = Promote home-monitoring of BP and behaviour changes associated with hypertension self-management  Con = Omron app - Blood pressure tracking app + home blood pressure monitor, plus routine care as prescribed by their regular clinicians | Self-report (unspecified questionnaire): MVPA mins.week^-1^  Baseline, 6-months | Goal setting (behaviour), problem solving, goal setting (outcome), action planning, discrepancy between current behaviour and goal, monitoring of behaviour by others without feedback, feedback on behaviour, self-monitoring of behaviour, self-monitoring of outcome(s) of behaviour, biofeedback, social support (emotional), information about health consequences, prompts/cues, social reward, adding objects to the environment | Outcome measure: MVPA mins.week^-1^  Effect size: 0.212  Standard error: 0.117 |
| Requena, 2019, Stroke, 50(7):1819-1824    Non-RCT, Spain | n = 159 (Exp 107, Con 52)  Age (yr) = 58.4 (SD 11.4)  F (%) = 44%  Dx = Stroke  Setting = Community dwelling following discharge home | 3-4-weeks  Target behaviour = PA + medication adherence  Exp = Farmalarm app for secondary prevention of stroke through vascular risk factor control = register obs + GPS + education + communicate with medical staff  Intensity = Medication alarm reminder at each time it is due. Contact with stroke team could be scheduled as requested by the participant  Purpose = Increase stroke awareness by medication alerts, chat communication with medical staff, didactic video files and exercise monitoring  Con = Usual care – stroke unit 90 day follow-up visit | Self-report (unclear): achieving >30 mins.day^-1^  90 days | Problem solving, monitoring of behaviour by others without feedback, self-monitoring of behaviour, monitoring of outcome(s) of behaviour without feedback, biofeedback, social support (unspecified), instruction on how to perform the behaviour, information about health consequences, prompts/cues, graded tasks, credible source, adding objects to the environment | Outcome measure: Proportion of group achieving 30-min exercise.day-1  Effect size: -0.008  Standard error: 0.324 |
| Salvi, 2018, J Telemed Telecare, 24(4):303‐316    Multicenter RCT, Spain, Germany and UK | n = 118 (Exp 55, Con 63)  Age (yr) = 58 (SD 10)  F (%) = 11%  Dx = CR graduates with CAD  Setting = Community dwelling following completion of phase-II CR | 21-weeks  Target behaviour = PA  Exp = HeartCycle GEx system available on smartphone and tablet = Mobile Station + Patient Station + Professional Station  Intensity = Automatic feedback generated by the system with on average 163 messages sent to each participant  Purpose = Motivate patients to adhere to their rehabilitation program through exercise monitoring, guidance, motivational feedback and educational content  Con = Usual care – Phase 3 standard rehabilitation according to the national procedures of each of the 3 countries. Also asked to report on daily PA on a paper diary | Self-report (custom questionnaire): MPA mins.week^-1^  Baseline, 6-months | Action planning, monitoring of behaviour by others without feedback, feedback on behaviour, self-monitoring of behaviour, self-monitoring of outcome(s) of behaviour, monitoring of outcome(s) of behaviour without feedback, biofeedback, feedback on outcome(s) of behaviour, instruction on how to perform the behaviour, information about health consequences, information about social and environmental consequences, prompts/cues, behavioural practice/rehearsal, graded tasks, credible source, social reward, adding objects to the environment, framing/reframing, incompatible beliefs | Outcome measure: MVPA mins.week^-1^  Effect size: 0.322  Standard error: 0.521 |
| Sengupta, 2020, JMIR Form Res, 4(6):e16420    Pre-Post, USA | n = 10  Age (yr) = 64.4 (SD 6.3)  F (%) = 100%  Dx = ACS or coronary revascularization in the last 10 years  Setting = Outpatient cardiology clinic | 12-weeks  Target behaviour = PA + diet  Exp = HerBeat app for smartphone and smartwatch = collect data on daily PA, HR, eating episodes, mood + personalised messages within the app  Intensity = Weekly encouragement to engage with the app  Purpose = To help women with behavioural self-management through goal-setting, tracking progress and educational videos  Con = N/A | Self-report (International Physical Activity Questionnaire-Short Form): days of MPA, MPA mins.day^-1^, sitting mins.day^-1^, days walked at least 10mins.day^-1^  Baseline, 12-weeks | Goal setting (behaviour), discrepancy between current behaviour and goal, feedback on behaviour, self-monitoring of behaviour, self-monitoring of outcome(s) of behaviour, biofeedback, feedback on outcome(s) of behaviour, social support (unspecified), social support (practical), social support (emotional), instruction on how to perform the behaviour, monitoring of emotional consequences, demonstration of the behaviour, prompts/cues, habit reversal, graded tasks, credible source, social reward, adding objects to the environment | N/A |
| Song, 2020, J Cardiovasc Transl, 13(4):659-667    RCT, China | n = 106 (Exp 53, Con 53)  Age (yr) = Exp 54.17 (SD 8.76), Con 54.83 (SD 9.13)  F (%) = 13.5%  Dx = Stable CHD  Setting = Community dwelling | 6-months  Target behaviour = PA  Exp = Telemonitoring software called MEMRS-CRS and WeChat app = HR monitoring (with belt) during exercise + personalised feedback  Intensity = Weekly feedback  Purpose = Provide remote CR through telemonitoring including exercise (following FITT principle) and vital signs  Con = Usual care – routine discharge education and outpatient follow-up with advice to exercise regularly  Both = Routine discharge education and outpatient follow-up which included advice to exercise regularly | Self-report (unclear): exercise habits according to meeting the American College of Sports Medicine 10^th^ edition of exercise testing and prescription  Baseline, 6-months | Feedback on behaviour, self-monitoring of behaviour, self-monitoring of outcome(s) of behaviour, monitoring of outcome(s) of behaviour without feedback, biofeedback, feedback on outcome(s) of behaviour, social support (unspecified), social support (emotional), credible source, adding objects to the environment | Outcome measure: Proportion of group meeting ACSM guidelines  Effect size: -0.003  Standard error: 0.688 |
| Weerahandi, 2020, JMIR Form Res, 4(5):e13989    Pre-Post, USA | n = 17  Age (yr) = 59 (SD 6)  F (%) = 60%  Dx = Hypertension  Setting = Community dwelling | 120-days  Target behaviour = Hypertension control – PA, diet, weight, BP measurement  Exp = DASH Mobile app + track diet, blood pressure, weight, PA + human coach and coach-facing web-based portal  Intensity = Coach interaction once per week via instant message, SMS or email  Purpose = Track diet, BP, weight, daily PA and coaching for improved hypertension management  Con = N/A | Device-measured (wireless pedometer): steps.day^-1^  Baseline (days 1-7), follow-up (days 46-120) | Goal setting (behaviour), problem solving, monitoring of behaviour by others without feedback, feedback on behaviour, self-monitoring of behaviour, self-monitoring of outcome(s) of behaviour, monitoring of outcome(s) of behaviour without feedback, biofeedback, feedback on outcome(s) of behaviour, social support (unspecified), demonstration of the behaviour, credible source, non-specific reward, adding objects to the environment | N/A |
| Werhahn, 2019, ESC Heart Fail, 6(3):516-525    Pre-Post, Germany | n = 10  Age (yr) = 46.3 (SD 7.8)  F (%) = 40%  Dx = Newly diagnosed HF with reduced ejection fraction  Setting = Community following discharge from hospital | 2-months  Target behaviour = PA  Exp = Cardio patient monitoring platform (CPMP) for smartphone and Apple smartwatch + Physician's tablet  Intensity = Optional reminders for medication. Encouraged daily input of vital signs and activity monitoring  Purpose = Remote monitoring through collecting HF symptoms, medication adherence, PA data and vital signs  Con = N/A | Device-measured (smartphone - iPhone 6SE, Apple Inc., Cupertino, CA, USA,  iOS Versions 10.2.1–11.2.1 + smartwatch - Apple Watch 1st Gen., Apple Inc., watchOS Versions 3.1.1–4.2.2): steps.day^-1^  Baseline, 1-month, 2-months | Monitoring of behaviour by others without feedback, self-monitoring of behaviour, self-monitoring of outcome(s) of behaviour, monitoring of outcome(s) of behaviour without feedback, biofeedback, feedback on outcome(s) of behaviour, adding objects to the environment | N/A |
| Widmer, 2015, J Cardiovasc Transl, 8(5):283-292    Non-RCT, USA | n = 44 (Exp 25, Con 19)  Age (yr) = Exp 60.2 (SD 12.1), Con 70.4 (SD 9.9)  F (%) = 18.2%  Dx = PCI for ACS  Setting = Outpatient phase-II Mayo Clinic CR | 3-months  Target behaviour = CVD risk factors – PA, weight, BP measurement, diet, laboratory values  Exp = Personal Health Assistant = integrated and personalised interface that tracks, logs, educates and forms actionable tasks  Intensity = Daily reminders for height, weight, BP, lab values, PA and diet. Occasional email reminders if the participant had not logged in recently  Purpose = Tracks, logs, educates and forms actionable tasks for the user to improve health  Con = Usual care – Mayo Clinic phase II CR program for 36 sessions  Both = Standard Mayo Clinic CR program for 36 sessions (approximately 3 months) | Self-report (Health behaviour questionnaire): exercise mins.week^-1^  Baseline, 3-months | Goal setting (behaviour), action planning, feedback on behaviour, self-monitoring of behaviour, feedback on outcome(s) of behaviour, social support (unspecified), instruction on how to perform the behaviour, information about health consequences, demonstration of the behaviour, prompts/cues, credible source, social reward, adding objects to the environment | N/A |
| Widmer, 2017, Am Heart J, 188:65‐72    RCT, USA | n = 71 (Exp 37, Con 34)  Age (yr) = Exp 62.5 (SD 10.7), Con 63.6 (SD 10.9)  F (%) = 18.3%  Dx = PCI for ACS  Setting = Outpatient phase-II Mayo Clinic CR | 3-months  Target behaviour = PA + diet  Exp = Personal Health Assistant (digital health intervention) and web-based application = patients to report dietary and exercise habits throughout CR + educational information  Intensity = Asked participants to log in 3-4 times weekly  Purpose = Tracks, logs, educates and forms actionable tasks for the user to improve health  Con = Usual care – Mayo Clinic phase II CR program for 36 sessions  Both = Standard Mayo Clinic CR program for 36 sessions (approximately 3 months) | Self-report (unspecified questionnaire): exercise mins.week^-1^  Baseline, 3-months | Monitoring of behaviour by others without feedback, self-monitoring of behaviour, instruction on how to perform the behaviour, prompts/cues, credible source, adding objects to the environment | Outcome measure: MVPA mins.week^-1^  Effect size: 0.455  Standard error: 0.241 |

Abbreviations: ACS, Acute coronary syndrome; ACSM, American College of Sports Medicine; App, Application; CR, Cardiac rehabilitation; CVD, Cardiovascular disease; Con, Control group; CABG, Coronary artery bypass graft surgery; CAD, Coronary artery disease; CHD, Coronary heart disease; Dx, Diagnosis; Exp, Experimental group; F, Female; HF, Heart failure; LPA, Light-intensity physical activity; MPA, Moderate-intensity physical activity; MVPA, Moderate-to-vigorous intensity physical activity; MI, Myocardial infarction; NS, Non-significant; PCI, Percutaneous coronary intervention; PAD, Peripheral artery disease; PA, Physical activity; RCT, Randomised control trial; SB, Sedentary behaviour; SD, Standard deviation.
