# Supplementary material for: Behaviour change techniques in cardiovascular disease smartphone apps to improve physical activity and sedentary behaviour: Systematic review and meta-regression
Source: Int J Behav Nutr Phys Act. 2022 Jul 7;19:81. doi: 10.1186/s12966-022-01319-8 (PMC9261070; doi:10.1186/s12966-022-01319-8)
Supplement: Supplementary file 2 — Additional file 2: Supplement 2. Behaviour change technique descriptions and examples from included studies [file 12966_2022_1319_MOESM2_ESM.docx]

**Supplement 2: Behaviour change technique descriptions and examples from included studies**

| **Behaviour change technique** | **Description and example** |
| --- | --- |
| **Feedback and monitoring** | |
| Monitoring of behaviour by others without feedback | Observe or record behaviour with the person’s knowledge as part of a behaviour change strategy.  Example: Monitoring of activity data including daily step count and heart rate via another app without providing feedback (Werhahn, 2019, ESC Heart Fail, 6(3):516-525). |
| Feedback on behaviour | Monitor and provide feedback on performance of the behaviour (e.g. form, frequency, duration, intensity).  Example: Coaching via two-way communication system where feedback could be customised based on the participants real-time data (Persell, 2020, JAMA Network Open, 3(3):e200255). |
| Self-monitoring of behaviour | A method for the person to monitor and record their behaviours as part of a behaviour change strategy.  Example: Ability to monitor physical activity (e.g. steps and activity minutes) in real-time via the app and a Fitbit (Freene, 2020, JMIR Form Res, 4(11):e17359). |
| Self-monitoring of outcome(s) of behaviour | Establish a method for the person to monitor and record an outcome of their behaviour.  Example: Blood pressure monitoring device connected with a smartphone to monitor changes as an outcome of a lifestyle management program involving physical activity (Kim, 2016, JMIR, 18(6):e116). |
| Monitoring of outcome(s) of behaviour without feedback | Observe or record outcomes of behaviour with the person’s knowledge.  Example: Blood pressure recordings uploaded to the smartphone were sent to the associated nurses to view but no feedback provided (Kim, 2016, JMIR, 18(6):e116). |
| Biofeedback | Provide feedback about the body in terms of physiological or biochemical state using an external monitoring device.  Example: The application displays heart rate monitoring during the participants activities (Grau-Pellicer, 2019, Top Stroke Rehabil, 27(5):354-368). |
| Feedback on outcome(s) of behaviour | Monitor and provide feedback on the outcome of performance of the behaviour.  Example: Feedback via an app push notification sent when participants attend new GPS locations as a result of being active in difference places (Freene, 2020, JMIR Form Res, 4(11):e17359). |
| **Goals and planning** |  |
| Goal setting (behaviour) | Agree to a goal defined in terms of the behaviour to be achieved.  Example: Setting and recording a personal goal for physical activity minutes per week (Johnston, 2016, Am Heart J, 178:85‐94). |
| Problem solving | Prompt the participant to analyse factors influencing the behaviour to generate and select strategies to overcome barriers.  Example: Communication with coach using motivational interviewing concepts elucidate relevant multilevel factors that may influence behaviour such as time, money, and community (Weerahandi, 2020, JMIR Form Res, 4(5):e13989). |
| Goal setting (outcome) | Set a goal defined in terms of a positive outcome of wanted behaviour.  Example: Setting a weight loss goal as an outcome of a health behaviour intervention (Lunde, 2020, Eur J Prev Cardiol, 27(16):1782-1792). |
| Action planning | Prompt the detailed planning of the behaviour including details of context, frequency, duration and intensity.  Example: Step count target increased by 10% at the end of each week if individuals achieved their step count target on five of seven days (Paul, 2016, Top Stroke Rehabil, 23(3):170‐177). |
| Review behaviour goal(s) | Review behaviour goals jointly with the person and consider modification such as re-setting the same goal, a small change to the goal or setting a new goal instead of the first.  Example: Activity prescription was reviewed if the participant did not reach the target and the health coach only progressed the target once the activity goal was achieved (Duscha, 2018, Am Heart J, 199:105-114). |
| Discrepancy between current behaviour and goal | Draw attention to any discrepancies between the person’s current behaviour and the person’s previously set goals.  Example: A progress function displaying the activity minutes goal and when the goal has not been met, it displays the number of remaining minutes to goal completion (Sengupta, 2020, JMIR Form Res, 4(6):e16420). |
| **Antecedents** |  |
| Restructuring the social environment | Change, or advise to change the social environment in order to facilitate performance of the behaviour.  Example: The app uses GPS coordinates and activity data to track and encourage participants to try change their social environment by visiting new GPS locations and increasing their activity levels (Freene, 2020, JMIR Form Res, 4(11):e17359). |
| Adding objects to the environment | Add objects to the environment in order to facilitate performance of the behaviour.  Example: Providing people with a smartphone app to use and/or a physical activity tracker such as a Fitbit. |
| **Repetition and substitution** | |
| Behavioural practice/rehearsal | Prompt practice or rehearsal of the behaviour in a context or at a time when the performance may not be necessary, in order to increase habit and skill.  Example: Push notification encouraging “On the hour, if you are sitting, get up and stretch, move for a couple of minutes” (Freene, 2020, JMIR Form Res, 4(11):e17359). |
| Behaviour substitution | Prompt substitution of the unwanted behaviour with a wanted behaviour.  Example: Push notification messages encouraging people to substitute sitting behaviours with more physically active behaviours (Freene, 2020, JMIR Form Res, 4(11):e17359). |
| Habit formation | Prompt rehearsal and repetition of the behaviour in the same context repeatedly so that the context elicits the behaviour.  Example: Sending push notification messages encouraging the person to get up and move every TV ad break (Freene, 2020, JMIR Form Res, 4(11):e17359). |
| Habit reversal | Prompt rehearsal and repetition of an alternative behaviour to replace an unwanted habitual behaviour.  Example: Push notification encouraging “On the hour, if you are sitting, get up and stretch, move for a couple of minutes” (Freene, 2020, JMIR Form Res, 4(11):e17359). |
| Generalisation of target behaviour | Advise to perform the wanted behaviour, which is already performed in a particular situation, in another situation.  Example: Commenced exercise program in a face-to-face setting and then gradually transitioned to repeating the exercises learnt, at home using the app (Nabutovsky, 2020, Israel Med Assoc J, 22(6):357-363). |
| Graded tasks | Set easy-to-perform tasks, making them increasingly difficult, but achievable, until behaviour is performed.  Example: Gradually increasing the step goal prescription every 4-weeks with intentions to build up to an overarching goal of increased daily physical activity (Duscha, 2018, Am Heart J, 199:105-114). |
| **Social support** |  |
| Social support (unspecified) | Provide social support (e.g. from friends, relatives, colleagues,’ buddies’ or staff), praise or reward for performance of the behaviour.  Example: Use of a messaging system which sends generalised support such as “Congratulations on meeting your steps goal this past week! Keep up the good work” (Lv, 2017, JMIR, 19(9):e311). |
| Social support (practical) | Advise on, arrange, or provide practical help (e.g. from friends, relatives, colleagues, ‘buddies’ or staff).  Example: Encouragement to walk with friends to support increased physical activity (Sengupta, 2020, JMIR Form Res, 4(6):e16420). |
| Social support (emotional) | Advise on, arrange, or provide emotional social support (e.g. from friends, relatives, colleagues, ‘buddies’ or staff).  Example: The app delivers personalised, gender-specific support in response to the participants behaviours and moods (Sengupta, 2020, JMIR Form Res, 4(6):e16420). |
| **Comparison of outcomes** | |
| Credible source | Present information from a credible source in favour of or against the behaviour.  Example: Using established guideline-based recommendations to tell patients what they need to accomplish with each goal in accordance with behaviour change theory (Widmer, 2015, J Cardiovasc Transl, 8(5):283-292). |
| **Natural consequences** |  |
| Information about health consequences | Provide information about health consequences of performing the behaviour.  Example: The app provides educational modules on exercise, weight management, smoking, and heart disease cause, symptoms and treatment (Johnston, 2016, Am Heart J, 178:85‐94). |
| Information about social and environmental consequences | Provide information about social and environmental consequences of performing the behaviour.  Example: Provides educational videos about the social and environmental consequences of not being physically active (Lv, 2017, JMIR, 19(9):e311). |
| Monitoring of emotional consequences | Prompt assessment of feelings after attempts at performing the behaviour.  Example: Provides opportunity to self-reflect on mood, effort, recovery, wellness and fatigue after physical activity via a questionnaire (Grau-Pellicer, 2019, Top Stroke Rehabil, 27(5):354-368). |
| **Associations** |  |
| Prompts/cues | Introduce environmental or social stimulus with the purpose of prompting or cueing the behaviour.  Example: If the participant has not set a physical activity goal by 4PM daily, a message prompting them to exercise was sent. If they were proactive in setting and achieving their walking goals, they were sent a positive reinforcing message (Sengupta, 2020, JMIR Form Res, 4(6):e16420). |
| Reduce prompts/cues | Withdraw prompts to perform the behaviour gradually.  Example: The weekly frequency of messages was initially higher with messages every second day for the first two weeks. Messages then reduced to three times per week (Johnston, 2016, Am Heart J, 178:85‐94). |
| **Shaping knowledge** |  |
| Instruction on how to perform the behaviour | Advise on how to perform the behaviour.  Example: mHealth platform includes education modules on physical activity and the health coach discusses and reaffirms the information (Duscha, 2018, Am Heart J, 199:105-114). |
| Information about antecedents | Provide information about events (e.g. social, environmental, emotional, cognition) that reliably predict performance of the behaviour.  Example: Data on risk factors and usual habits around physical activity and sedentary behaviour are collected through the app and then trigger push notification messages related to those negative habits (e.g. prolonged TV watching) and encouraging alter options to trigger healthier habits (Freene, 2020, JMIR Form Res, 4(11):e17359). |
| **Reward and threat** |  |
| Social reward | Arrange verbal or non-verbal reward if there has been effort and/or progress in performing the behaviour.  Example: The health coach sends congratulatory messages through the app when an activity goal is met and provides positive reinforcement messages when the participant is proactive in setting their walking goal (Sengupta, 2020, JMIR Form Res, 4(6):e16420). |
| Social incentive | Inform that a verbal or non-verbal reward will be delivered if there has been effort and/or progress in performing the behaviour.  Example: Informed participants that their fish alias will get bigger the more they meet their activity goals and that their peers will also be able to see this (Paul, 2016, Top Stroke Rehabil, 23(3):170‐177). |
| **Comparison of behaviour** | |
| Demonstration of the behaviour | Provide an observable sample of the behaviour, directly or indirectly via film or pictures.  Example: Educational videos and articles provided through the app related to the program and physical activity (Nabutovsky, 2020, Israel Med Assoc J, 22(6):357-363). |
| Social comparison | Draw attention to other people’s performance to allow comparison with their own.  Example: The app provides real time feedback on the person’s own physical activity and that of each of the other members in the group through use of a fish alias (Paul, 2016, Top Stroke Rehabil, 23(3):170‐177). |
| **Identity** |  |
| Framing/reframing | Suggest the deliberate adoption of a perspective or new perspective on behaviour in order to change cognitions or emotions about performing the behaviour.  Example: Prompting based on the health belief model to shape the desired behaviour of increased activity (Salvi, 2018, J Telemed Telecare, 24(4):303‐316). |
| Incompatible beliefs | Draw attention to discrepancies between current or past behaviours in order to create discomfort.  Example: Prompts thought about any misconceptions about the benefits of exercise via a questionnaire (Salvi, 2018, J Telemed Telecare, 24(4):303‐316). |
| **Regulation** |  |
| Conserving mental resources | Advise on ways of minimising demands on mental resources to facilitate behaviour change.  Example: The push notification messages through the app suggest small context-specific actionable micro behavioural alternatives to their usual behaviours so that each task is not a big change or requiring large mental demand, such as moving the rubbish bin a little further away to encourage more incidental walking (Freene, 2020, JMIR Form Res, 4(11):e17359). |
