# Supplementary material for: Behaviour change techniques in cardiovascular disease smartphone apps to improve physical activity and sedentary behaviour: Systematic review and meta-regression
Source: Int J Behav Nutr Phys Act. 2022 Jul 7;19:81. doi: 10.1186/s12966-022-01319-8 (PMC9261070; doi:10.1186/s12966-022-01319-8)
Supplement: Supplementary file 3 — Additional file 3: Supplement 3. Grid plot of behaviour change techniques and studies ranked by observed effect size [file 12966_2022_1319_MOESM3_ESM.docx]

**Supplement 4: Grid plot of behaviour change techniques and studies ranked by observed effect size**


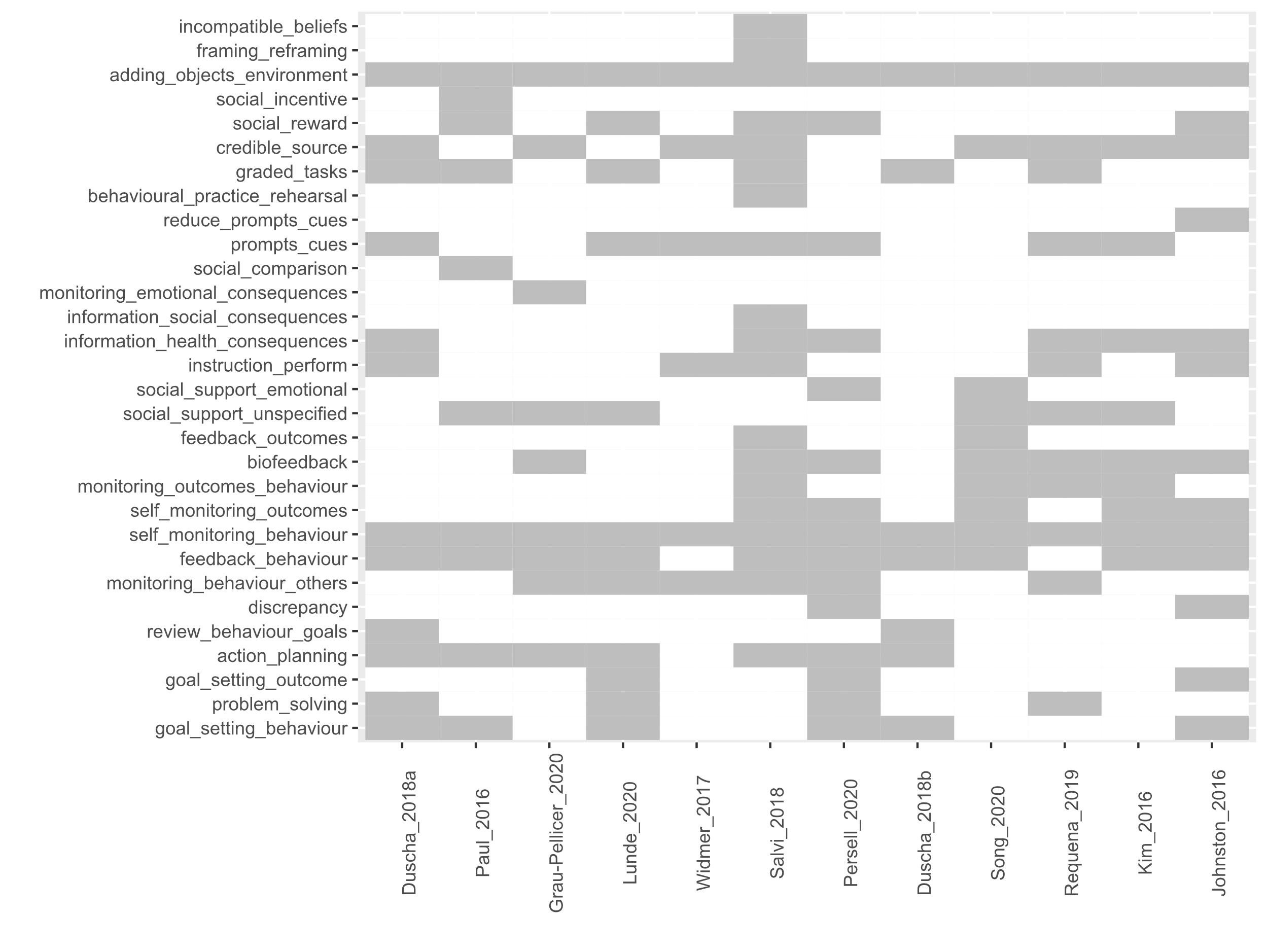


Note: Studies are ranked by observed effect size, decreasing from left to right.

Abbreviated BCT labels: incompatible_beliefs, incompatible beliefs; framing_reframing, framing/reframing; adding_objects_environment, adding objects to the environment; social_incentive, social incentive; social_reward, social reward; credible_source, credible source; graded_tasks, graded tasks; behavioural_practice_rehearsal, behavioural practice/rehearsal; reduce_prompts_cues, reduce prompts/cues; prompts_cues, prompts/cues; social_comparison, social comparison; monitoring_emotional_consequences, monitoring of emotional consequences; information_social_consequences, information about social and environmental consequences; information_health_consequences, information about health consequences; instruction_perform, instruction on how to perform the behaviour; social_support_emotional, social support (emotional); social_support_unspecified, social support (unspecified); feedback_outcomes, feedback on outcome(s) of behaviour; biofeedback, biofeedback; monitoring_outcomes_behaviour, monitoring of outcome(s) of behaviour without feedback; self_monitoring_outcomes, self-monitoring of outcome(s) of behaviour; self_monitoring_behaviour, self-monitoring of behaviour; feedback_behaviour, feedback on behaviour; monitoring_behaviour_others, monitoring of behaviour by others without feedback; discrepancy, discrepancy between current behaviour and goal; review_behaviour_goals, review behaviour goal(s); action_planning, action planning; goal_setting_outcome, goal setting (outcome); problem_solving, problem solving; goal_setting_behaviour, goal setting (behaviour).
